# Cubosomes with surface cross-linked chitosan exhibit sustained release and bioavailability enhancement for vinpocetine

**DOI:** 10.1039/c8ra10302j

**Published:** 2019-02-21

**Authors:** Yuanfeng Wei, Jianjun Zhang, Yazhen Zheng, Yaxiang Gong, Meng Fu, Chengran Liu, Liang Xu, Changquan Calvin Sun, Yuan Gao, Shuai Qian

**Affiliations:** School of Traditional Chinese Pharmacy, China Pharmaceutical University Nanjing 210009 China newgaoyuan@163.com silence_qs@163.com +86 25 83379418 +86 25 83379418 +86 139 15957175; School of Pharmacy, China Pharmaceutical University Nanjing 210009 China; Department of Pharmaceutics, College of Pharmacy, University of Minnesota Minneapolis MN 55455 USA

## Abstract

The present study aims to develop cubosomes with surface cross-linked chitosan for sustained drug delivery and enhanced oral bioavailability of vinpocetine (VPT). GMO based liquid cubosomes with VPT loading were prepared by the high pressure homogenization method. In order to enhance the anti-digestion effect, chitosan was cross-linked on cubosomes by the Schiff reaction, followed by solidification *via* spray drying. The obtained spray-dried cubosomes (chito-cubosomes) are spherical microspheres with nano-sized holes on the surface. After reconstitution, the particle size and zeta potential of chito-cubosomes were determined to be ∼250 nm and +35.9 mV, respectively. In comparison to unmodified liquid cubosomes, chito-cubosomes exhibited a significant anti-digestion effect with a typical sustained release profile. In comparison to a VPT suspension, liquid cubosomes showed a 2.5-fold higher *C*_max_ and 3.0-fold higher AUC_0–∞_, while chito-cubosomes further enhanced bioavailability (5.0-fold) with prolonged MRT (2.2-fold) and delayed *T*_max_ (2.8-fold). The results suggested that chito-cubosomes could be a promising drug carrier for enhancing oral absorption with sustained release behavior.

## Abstract 2

Cubosomes with surface cross-linked chitosan exhibit anti-digestion effect, sustained drug release behavior, and significantly enhanced oral bioavailability of vinpocetine.

## Graphical abstract

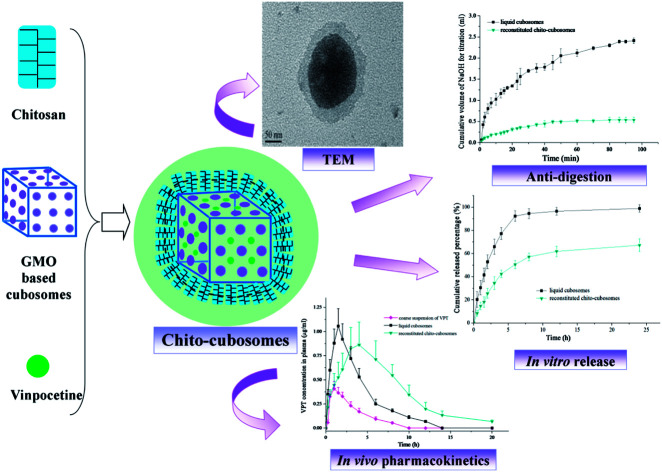

## 1. Introduction

**Fig. 1:**
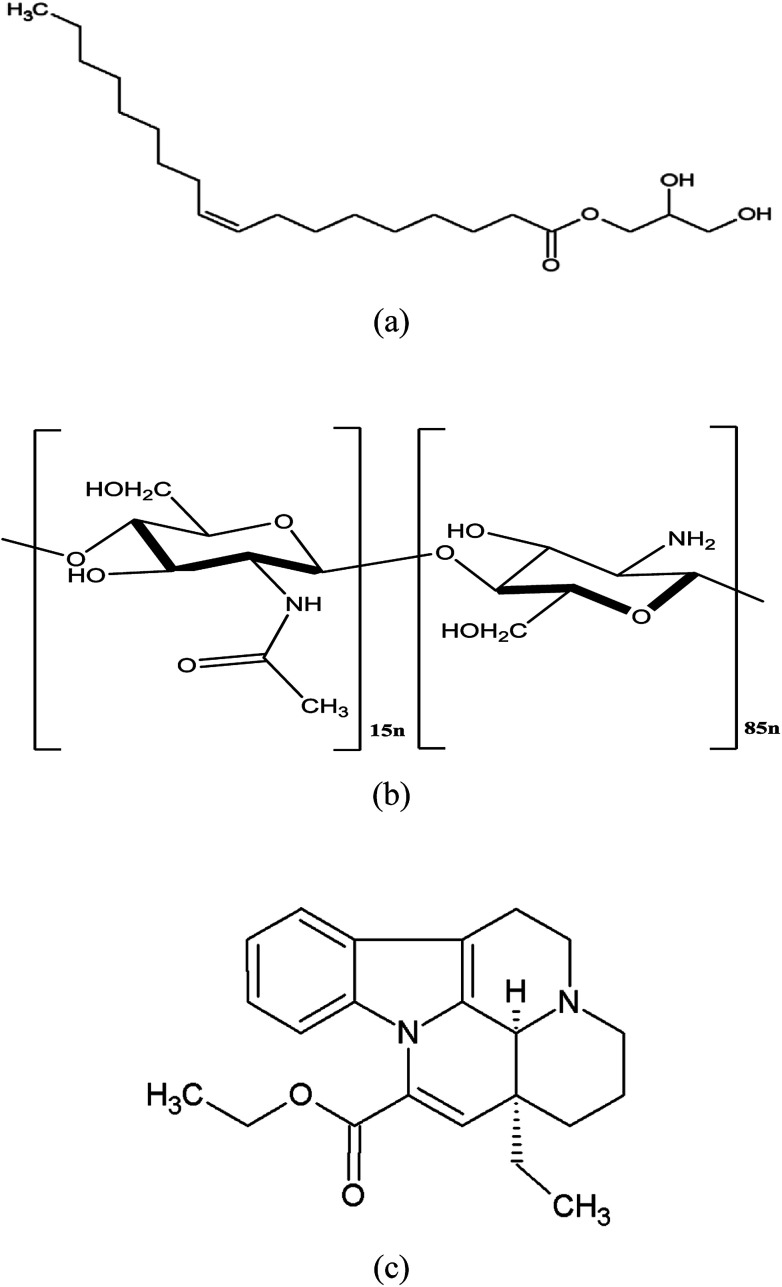
Chemical structure of (a) glycerol monooleate (GMO), (b) chitosan (CS) and (c) vinpocetine (VPT).

Glycerol monooleate (GMO, Fig. 1a) is a lipid molecule with an extraordinary ability to form different liquid crystalline phases, such as lamellar and hexagonal cubic, in aqueous media and biological fluids.^1^ These mesophases provide opportunities to modify drug release using lipid-based systems for both hydrophilic and hydrophobic drugs since their liquid crystalline structure can hinder drug release into the outer continuous phase.^2,3^ GMO-based cubosomes are bicontinuous cubic liquid crystal nanoparticles with the internal structure of a non-lamellar liquid crystal. Cubosomes are usually prepared by top-down methods, *e.g.*, high-pressure homogenization and ultrasonication, with suitable stabilizers to prevent the aggregation of particles.^4^ The commonly used stabilizers are bile salts, amphiphilic proteins, and block polymers (*e.g.* poloxamers). Compared to liposomes, cubosomes have advantages including ease of preparation, better physical stability, and special liquid crystalline properties.^5^ Although GMO-based cubosomes hold the promise for achieving sustained release *in vivo*, they are susceptible to degradation in the gastrointestinal tract catalyzed by lipase, which compromises liquid crystalline structure^6,7^ and, thereby, loss of the sustained drug release property *in vivo*.^8,9^

Chitosan (CS, Fig. 1b) is a natural linear polycationic polysaccharide derived from the alkaline deacetylation of chitin. It is a non-toxic and biodegradable polymer with the ability to form gel and micro- or nano- particles. Its biocompatibility makes it a popular carrier for drug delivery. CS is insoluble at neutral pH, but it is soluble and positively charged under acidic environment. Hence, it can strongly interact with negatively charged molecules through electrostatic force. Importantly, CS could be chemically cross-linked with a cross-linking agent (*e.g.* glutaraldehyde) through the Schiff reaction between the amino-groups of CS and aldehyde group of glutaraldehyde.^10^ In our previous study, CS was linked to the oxidized glycerol monooleate (OGMO) *via* imine bonds (–C=N–) to obtain a new CS-based polymer (OGMO-CS), which rapidly forms hydrogels in aqueous media to provide sustained release behavior.^11^ Recent studies demonstrated that the CS coated emulsions or liposomes could also possess sustained drug release properties.^12–14^ In addition, CS coated liposomes enhanced cellular uptake and decreased the cytotoxicity of anticancer drugs,^15^ and CS-encapsulated emulsion possessed stronger anti-lipase ability than the pure emulsion system.^10^ The aim of this study was to develop cubosomes with surface cross-linked CS which could prevent the digestion of cubosomes in intestinal tract, thereby, enabling the sustained drug release and further *in vivo* bioavailability enhancement.

Vinpocetine (VPT, Fig. 1c), a semi-synthetic derivative of vincamine, has been wildly used to treat acute stroke and senile cerebral dysfunction.^16,17^ However, poorly aqueous solubility and extensive first-pass metabolism are two major factors responsible for its low oral bioavailability (∼6%).^18,19^ The relatively low oral bioavailability associated with short *t*_1/2_ (∼1.3–2.5 h) severely limited its clinical efficacy.^20–22^ In this study, superficial CS cross-linked cubosomes encapsulating VPT (chito-cubosomes) was designed and prepared. *In vitro* digestion and release studies indicated that the prepared chito-cubosomes could not only have anti-digestion effect, but also exhibit sustained release behavior. In addition, *in vivo* pharmacokinetic evaluation was also performed.

## 2. Materials and methods

### 2.1. Materials

Vinpocetine was purchased from Kailun New Chemical Materials Limited (Wuhan, China). Glycerol monooleate was a gift from Danisco Cultor (Grindsted, Denmark). Poloxamer 407 was provided by BASF (Ludwigshafen, Germany). Sephadex G-50 was purchased from Pharmacia (Uppsala, Sweden). Chitosan (50 kDa) with a deacetylation degree of 85% was obtained from Jinan Haidebei Marine Bioengineering Co., Ltd. (Jinan, China). Glutaraldehyde lipase (from porcine pancreas) and bile extract (porcine) were obtained from Sigma-Aldrich (Poole, UK). Progesterone (as internal standard) was purchased from Aladdin Chemistry Co. Ltd. (Shanghai, China). HPLC grade water was produced by a Milli-Q water purification system (Millipore Co., Ltd., Bedford, USA). All other chemicals were obtained from Sinopharm Chemical Reagent Co. Ltd. (Shanghai, China).

### 2.2. Preparation of liquid cubosomes

Cubosomes were prepared by high-pressure homogenization as previously described.^23^ Briefly, 75 mg of VPT was dissolved in 2.5 g of molten GMO at 37 °C. The obtained solution was added dropwise into 23 mL of poloxamer 407 solution (1.5%, w/v) under magnetic stirring at 37 °C, followed by high-pressure homogenization under 1200 bar for 5 cycles using a homogenizer (Avestin Emulsiflex-C5, Avestin Inc., Ottawa, Canada). The concentration of VPT in the obtained liquid cubosomes was about 3 mg mL^−1^.

### 2.3. Preparation of spray-dried chito-cubosomes

CS solution was prepared by dissolving 2 g CS in 25 mL of 3% citric acid aqueous solution. The obtained solution was slowly added into 25 mL of above prepared liquid cubosomes under magnetic stirring at 200 rpm at 25 °C. After stirring for 10 min, 0.4 or 0.8 mL of glutaraldehyde solution (6.25%, w/v) was then added into the mixture to initiate the cross-linking reaction between CS and glutaraldehyde. The reaction was allowed to continue for 4 h.^24^

After cross-linking reaction, 2 g of mannitol as scaffolds was dissolved in the obtained reaction mixture by stirring, followed by spray dryer (Büchi B-290: Büchi Labortechnik AG, Switzerland). The spray drying was carried out using a 0.7 mm nozzle with the inlet temperature of 110 °C, aspirator setting of 40 m^3^ h^−1^, spray flow rate of 600 L h^−1^, pump setting of 2.72 mL min^−1^. The spray-dried chito-cubosomes solid was collected and stored in desiccators at 25 °C for further characterizations.

### 2.4. Physicochemical characterizations

#### 2.4.1. Particle size and zeta potential

The particle size and zeta potential of liquid cubosomes and chito-cubosomes were determined by photon correlation spectroscopy (PCS) using a Zetasizer 3000 (Malvern Instruments, Malvern, UK) at 25 °C. The measurements yield the volume weighted mean particle size, the polydispersity index (PI) and zeta potential. All samples were analyzed in triplicates.

#### 2.4.2. Atomic force microscopy

Atomic force microscopy (AFM) (Veeco, diNanoScope V, USA) was used to characterize the morphology as well as visualized particle size of VPT-loaded cubosomes. A drop of properly diluted cubosomes was placed on the surface of a clean silicon wafer. The sample was then scanned in contact mode to record images.

#### 2.4.3. Scanning electron microscope

Scanning electron microscope (SEM, Hitachi S3400, Tokyo, Japan) was applied to study the surface morphology of VPT loaded chito-cubosomes powder. Samples were glued and mounted on metal sample plates. The samples were gold coated (thickness ≈ 15–20 nm) with a sputter coater (Fison Instruments, UK) using an electrical potential of 2.0 kV at 25 mA for 10 min. An excitation voltage of 20.0 kV was used in the experiments.

#### 2.4.4. Transmission electron microscopy

Transmission electron microscopy (TEM, JEOL-200CX, Tokyo, Japan) was employed to study the interior structure of liquid cubosomes and reconstituted chito-cubosomes. A drop of cubosomes was placed onto a carbon-coated copper grid, excess amount was removed with a filter paper leaving a thin liquid film on the holes. The films were negatively stained with phosphotungstic acid solution (1.5% w/w, pH 6.5). Finally, the stained samples were dried under an infrared lamp. The liquid mixture containing CS and liquid cubosomes without adding the cross-linker glutaraldehyde was also visualized under TEM to study the effect of CS on the structure of cubosomes.

#### 2.4.5. Thermogravimetric analysis

Residual solvent present in the spray dried chito-cubosomes was examined by a thermogravimetric analyzer (TG 209 F3 Tarsus®, Netzsch, Selb, Germany). Samples were loaded in aluminum pans and heated from 30 to 300 °C at a rate of 10 °C min^−1^ in nitrogen atmosphere with a purge rate of 20 mL min^−1^. Data analysis was performed using NETZSCH-Proteus software (version 4.2).

### 2.5. Drug loading determination

The drug loading capacity of the liquid crystalline phase described here for VPT was evaluated by determination of the equilibrium solubility of VPT in GMO as the method previously reported.^25,26^ Excess amount of VPT powder was dispensed into 5 mL of molten GMO at 37 °C, and the mixture was stirred until the equilibrium between solid and liquid achieved. VPT content was analyzed on a HPLC system with suitable dilution by mobile phase. Chromatographic separation was performed on a Shimadzu LC-2010AHT HPLC system with a Kromasil C_18_ column (250 mm × 4.6 mm i.d., 5 μm particle size). The mobile phase consisting of methanol and 10 mM ammonium carbonate solution (95/5, v/v) was run at 1.0 mL min^−1^ with the column temperature at 30 °C. The detection wavelength was set at 274 nm. To compare the relative solubility enhancement, the solubilities of VPT in pure water and phosphate buffer with pH 6.5 were determined. The solubilization enhancement ratio (SER) was obtained by the following equation:^25^SER = solubility_phase_/solubility_buffer_where solubility_phase_, solubility_buffer_ denoted the drug solubility in the liquid crystalline phase and buffer solution, respectively.

### 2.6. Entrapment efficiency determination

The entrapment efficiency of VPT in cubosomes was determined by gel permeation chromatography using Sephadex G-50 as previously described.^27^ In this study, a mini-column of Sephadex G-50 (*Φ* 1.0 cm × h 6.0 cm) was used to separate free VPT from encapsulated VPT in cubosomes. The spray-dried chito-cubosomes were reconstituted in water before loading, while the liquid cubosomes generated from high-pressure homogenizer were directly loaded. After preconditioning with 2.5 mL distilled water, 2.5 mL of cubosomes was loaded on Sephadex G-50 column, then the system was centrifuged (25 °C, 4000 × *g*) for 1 min, and the centrifugate was collected. In order to completely wash out residual on the surface of Sephadex G-50 gel, another 2.5 mL of distilled water was then added on the column and centrifuged under same condition. To determine the amount of VPT entrapped in cubosomes, VPT content in combined centrifugate (*i.e.* free VPT) and total VPT in liquid cubosomes or chito-cubosomes before loading on the column (dissolved and diluted by methanol) were analyzed by HPLC-UV method mentioned in Section 2.5.

The entrapment efficiency (EE%) of cubosomes was calculated by the following equation:EE% = (*W*_total_ − *W*_centrifugate_)/*W*_total_ × 100%Where *W*_centrifugate_ and *W*_total_ denoted the free VPT in centrifugate and the total drug in chito-cubosomes before loading on the column, respectively.

### 2.7. *In vitro* digestion of VPT cubosomes and chito-cubosomes

A modified pH-stat titration method was employed to study the anti-digestion ability of cubosomes and spray-dried chito-cubosomes.^9,29^ The experiments were performed at 37 °C under continuous stirring. Either VPT liquid cubosomes or spray-dried chito-cubosomes (equivalent to 1 gram of GMO) were dispersed in 30 mL of purified water. 5.0 mL of bile extract (37.5 mg mL^−1^ in 0.01 M phosphate buffer, pH 7.0) and 1.0 mL of CaCl_2_ (27.5 mg mL^−1^ in the same buffer) were then added and pH was adjusted to 7.0 with 1 M NaOH. After adding 1.5 mL of lipase solution (40 mg mL^−1^ in PBS 7.0), the pH of the digestion solution was titrated with 0.01 M NaOH using a buret to a pH 7.0 at different time intervals from 1 min to 90 min. The amount of consumed 0.01 M NaOH equals to the amount of released free fatty acids during the GMO lipolysis process.

### 2.8. *In vitro* release study

*In vitro* release of VPT from liquid cubosomes and spray-dried chito-cubosomes were carried out at 37 °C using the dynamic dialysis method.^30,31^ 0.1 M HCl was used as the release medium to achieve the sink condition for the release of VPT. About 1.5 g of spray-dried chito-cubosomes were reconstituted in 5 mL of distilled water. 1.5 mL of the reconstituted chito-cubosomes or liquid cubosomes was sealed in dialysis bag (MWCO: 8000–12 000 Da) (*n* = 3). VPT dissolved in 0.1 M HCl (containing 2% Tween 80) at 3 mg mL^−1^ was used as control. Dialysis bags after sample loading were immersed in 1000 mL of medium. The paddle rotating speed was 100 rpm. At predetermined time intervals, 5 mL of medium were withdrawn and filtered with 0.22 μm PTFE membrane prior to HPLC analysis. After each sampling, the same volume of fresh medium was immediately supplied to the dissolution vessel.

### 2.9. *In vivo* absorption study

#### 2.9.1. Animals

Male Sprague-Dawley rats (220 ± 10 g) were obtained from the Laboratory Animal Center, China Pharmaceutical University (Nanjing, China). Animals were housed in standard cages on a 12 h light–dark cycles, fed with standard animal chow and tap water daily. All animals used in this study were handled in accordance with the guidelines of the Principles of Laboratory Animal Care (State Council, revised 1988). The study was approved by the Ethical Committee of China Pharmaceutical University.

One day before the pharmacokinetic study, each animal was operated with a cannula insert into the right jugular vein under anesthesia by intraperitoneal injection of pentobarbital sodium (50 mg kg^−1^). A surgical incision was made on the ventral side of the neck of rats to expose the jugular vein. The jugular vein was then cannulated with a polyethylene tube (0.5 mm ID, 1 mm OD, Portex Ltd., Hythe, Kent, England) that was led under the skin and exteriorized at the back of the neck for blood sampling. 50 IU mL^−1^ of heparin sodium in normal saline was filled into the catheter to prevent the blood clotting.^32^ After the exposed areas were surgically sutured, the rats were placed individually in standard cages. The animals were allowed to recover for 24 hours and were fasted overnight prior to drug administration.

#### 2.9.2. Drug administration

Before gavage administration to the rats, coarse suspension was prepared by dispersing powder of crystalline VPT in sodium carboxymethyl cellulose aqueous solution (0.5%, w/v). Spray-dried chito-cubosomes were reconstituted in water with VPT concentration of 2 mg mL^−1^.

The rats were randomly divided into three groups (five rats in each group) and orally administrated with coarse suspension of crystalline VPT, liquid cubosomes, and reconstituted chito-cubosomes at a VPT dose of 10 mg kg^−1^, respectively. After gavage administration, about 250 μL of blood sample was collected from the jugular vein into heparinized tubes at 0, 0.25, 0.5, 1, 1.5, 2, 3, 4, 6, 8, 10, 12, 14 and 20 h. Plasma was separated by centrifugation (10 °C, 10 000 × *g*, 15 min) using a refrigerated table top centrifuge (Sigma 1–15K, Sigma, Germany) and kept frozen at −20 °C until analysis.

#### 2.9.3. Analysis of VPT in rat plasma

Frozen plasma samples were thawed at room temperature just before sample preparation. 100 μL of rat plasma was mixed with 10 μL of progesterone solution (internal standard, 48.1 μg mL^−1^ in methanol) and 10 μL of 5 M NaOH. After vortexing for 2 min, the plasma samples were extracted with 1 mL diethyl ether by vortex-mixing for 10 min and centrifuged for 5 min (10 °C, 10 000 × *g*). Ether phase was combined and evaporated to dryness under nitrogen stream at 35 °C. The solid residue was reconstituted by 100 μL of methanol. After centrifugation for 5 min (10 °C, 10 000 × *g*), an aliquot of 20 μL supernatant was injected into a reversed phase HPLC (Shimadzu 2010AHT, Shimadzu Corporation, Kyoto, Japan). Chromatographic separation of VPT was performed on a Phenomenon Luna ODS C_18_ column (250 mm × 4.6 mm, 5 μm). Mobile phase consisting of methanol and 0.1 M ammonium carbonate aqueous solution with a ratio of 88/12 (v/v) was run at 1.2 mL min^−1^ and monitored at 274 nm. The column temperature was set at 30 °C.

Under the current developed HPLC-UV method, VPT peak could be baseline separated from internal standard with no interference from endogenous materials in rat plasma. Good linearity was obtained for VPT over the concentration range of 50–8000 ng mL^−1^ (*r*^2^ > 0.995, *n* = 6). The limit of quantification and limit of detection for VPT were determined to be 35.5 and 5.4 ng mL^−1^, respectively. At concentrations of 50, 1000 and 8000 ng mL^−1^, spiked recoveries of VPT from rat plasma were 85.2%, 88.7% and 90.4%, respectively (*n* = 3); the relative standard deviation (RSD) of both inter-day and intra-day precision was below 10%; and the accuracy was less than 10% relative error (RE). There was no detectable degradation or loss of VPT after storage for two weeks at −20 °C and freeze-thawing for three cycles.

#### 2.9.4. Data analysis

The plasma concentration–time profiles of each rat were analyzed by non-compartmental method using PKSolver software (issued by China Pharmaceutical University).^33^ The maximum plasma concentration (*C*_max_) and the time to reach maximum plasma concentration (*T*_max_) were directly obtained from plasma data. The area under the plasma concentration–time curve from time 0 to 20 h (AUC_0–20 h_) and from time 0 to infinity (AUC_0–∞_) were calculated by the trapezoidal method without or with extrapolation to time infinity (AUC_0–∞_ = AUC_0–20 h_ + *C*_20 h_/*λ*_z_), where *λ*_z_ was the first-order rate constant associated with the terminal (log-linear) portion of the curve. Mean residence time (MRT) was calculated by AUMC/AUC, where AUMC was the area under the curve of concentration multiplies time *versus* time. Relative bioavailability (RB, %) was calculated by AUC_0–∞ (test)_/AUC_0–∞(coarse suspension of VPT)_ × 100%.

All results were expressed as mean ± S.D. Statistical data analyses were performed using one-way analysis of variance (ANOVA) with *p* < 0.05 as the minimal level of significance.

## 3. Results and discussion

### 3.1. Optimization of poloxamer 407 concentration and homogenization parameters

To produce homogenous cubosomes and reduce the presence of micron-sized particle aggregates, high-pressure homogenization was employed. In the composition of cubosomes, poloxamer 407 was used to provide steric stabilization against coalescence and/or agglomeration of cubosomes, by anchoring its polypropylene oxide blocks in the polar region or at the surface of GMO bilayer.^34,35^ To optimize the preparation process of VPT loaded cubosomes, the effect of poloxamer 407 concentration, the homogenization pressure, and number of homogenization cycle were investigated with particle size and its distribution as responses.

**Fig. 2:**
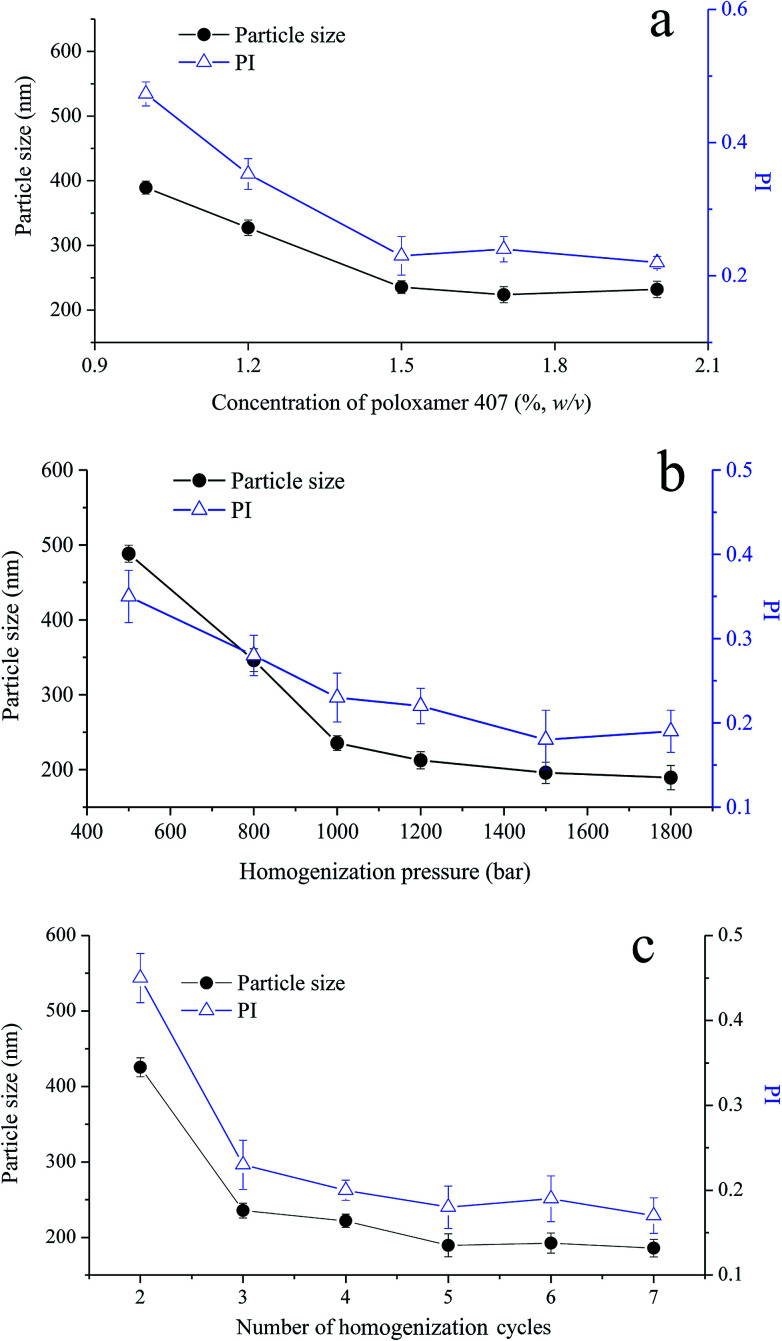
Effect of (a) concentration of poloxamer 407 (1000 bar, 3 cycles); (b) homogenization pressure (3 cycles); and (c) number of homogenization cycles (1200 bar) on particle size and polydispersity index (PI) of VPT-loaded liquid cubosomes.

Under three cycles homogenization at a pressure of 1000 bar, the initial cubosomal particle size of 400 nm decreases with the increasing concentration of poloxamer 407 until reaching ∼230 nm at the concentration of 1.5%, beyond which no further particle size reduction was observed (Fig. 2a). Particle distribution reflected as polydispersity index (PI) showed a similar trend. Thus, 1.5% poloxamer was selected in the formulation of cubosomes. In addition, an increase of either homogenization pressure (Fig. 2b) or cycle numbers (Fig. 2c) lead to a decrease of the particle size to around 200 nm with a PI of about 0.2. The appearance of cubosomes changed from milky emulsion-like to opalescent dispersion while increasing pressure from 500 bar to 1000 bar, showing a significant decrease of particle size and its PI.^36^ According to above optimization, homogenization parameters were set as 1200 bar pressure and 5 cycles. Under this condition, the particle size and PI of VPT loaded liquid cubosomes were 189.5 ± 15.2 nm and 0.19 ± 0.03, respectively.

### 3.2. Physicochemical characterizations

#### 3.2.1. Particle size and zeta potential

**Fig. 3:**
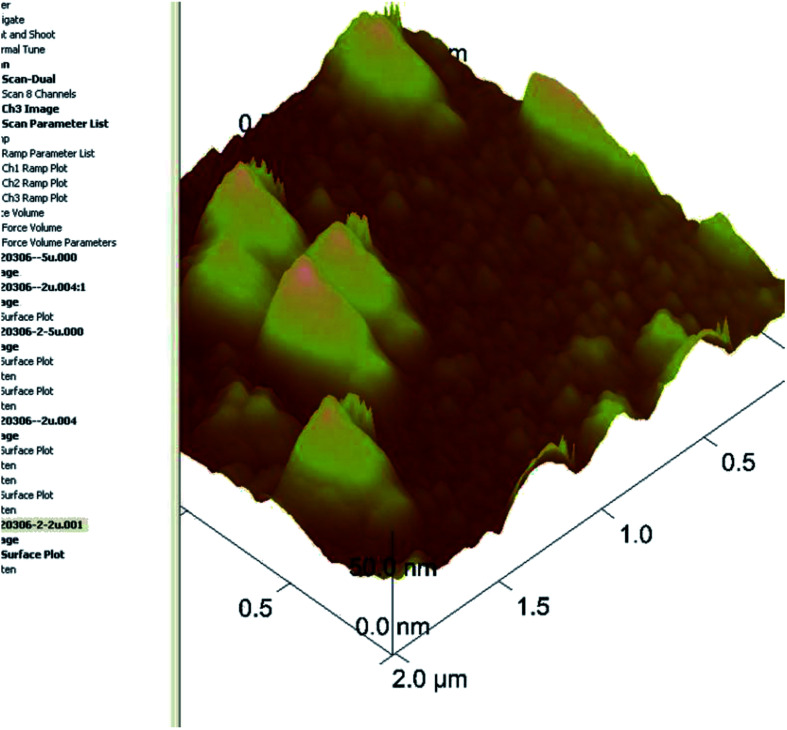
AFM image of liquid cubosomes.

Since AFM could image surfaces immersed in a liquid, it is more suitable for revealing the morphology of soft matter like cubosomes than traditional cryo-TEM, where the freezing process might affect the morphology of original cubosomes.^37^ An AFM image of VPT loaded liquid cubosomes on the surface of the silicon wafer is shown in Fig. 3. The apparent height and particle size of cubosomes was about 50 nm and 350 nm, respectively. The larger particle size observed by AFM than that determined by PCS (∼189.5 nm) might be due to flattening effect of GMO based cubosomes on the wafer surface.^37^ After surface cross-linking with CS and spray drying, the particle size of reconstituted chito-cubosomes was determined to be 246.5 ± 45.7 nm with a PI of 0.35 by PCS.

Zeta potential is an important physicochemical parameter that influences the physical stability of colloidal systems. Generally, a colloidal system with zeta potential above +30 mV or below −30 mV is considered to be stable.^38,39^ In our study, zeta potential of the prepared VPT loaded liquid cubosomes was determined to be −21.5 mV. The negative charge of liquid cubosomes could be due to the trace amount of free oleic acid existed in commercial GMO.^23^ However, after surface modification with CS and crosslinking by glutaraldehyde, the reconstituted chito-cubosomes reversed to positive charge with a zeta potential of +35.9 mV. Such change should be ascribed to the protonation of positive charged CS.^40^

#### 3.2.2. TEM

**Fig. 4:**
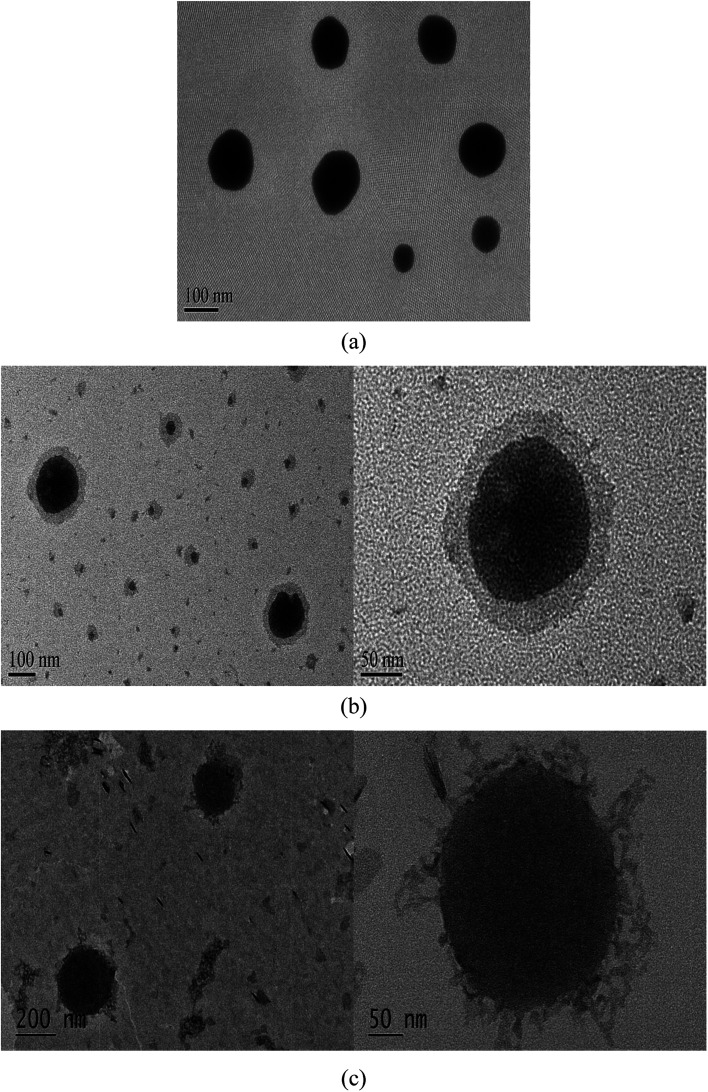
TEM of (a) liquid cubosomes, (b) physical mixture of CS solution and liquid cubosomes, and (c) reconstituted chito-cubosomes.

TEM experiments were performed to observe the morphology of liquid cubosomes, physical mixture of liquid cubosomes and CS without cross-linking, and reconstituted spray-dried chito-cubosomes dispersions in wet state. As shown in Fig. 4a, liquid cubosomes tended to form spherical nanoparticles with the size of around 120 nm. After adding CS solution in liquid cubosomes, a semi-transparent outer shell covered on the dark cubosomal core could be clearly observed with a thickness of about 30 nm (Fig. 4b). This might be due to electrostatic adsorption of positive charged CS on negative charged cubosomes, and agreed with the reversal of zeta potential from liquid cubosomes and chito-cubosomes as described above. The surface cross-linked CS membrane has also been applied for function modification of silver nanoparticles,^41,42^ catalase^43^ and PLGA nanoparticles.^44^Fig. 4c displays the morphology of reconstituted spray-dried chito-cubosomes. The particle size of reconstituted chito-cubosomes was around 250 nm. In comparison to the physical mixture without cross-linking, the out layer of chito-cubosomes seemed rough and branched, which might be due to the cross-linking reaction of CS with glutaraldehyde.

#### 3.2.3. SEM and TGA for chito-cubosomes

**Fig. 5:**
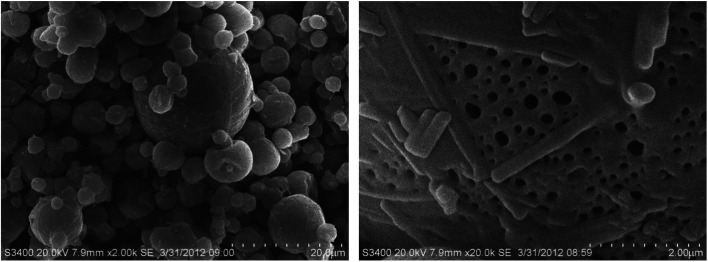
SEM of spray dried chito-cubosomes.

SEM was employed to visualize the morphology of spray-dried chito-cubosomes (Fig. 5). They displayed as spherical shape with particle size of ∼5–20 μm. The surface of chito-cubosomes seemed smooth in large visual field (left figure), but nano-sized holes scattered on the entire surface could be visualized in greater magnification (right figure). These holes might facilitate the entry of the dissolution medium for reconstitution.

**Fig. 6:**
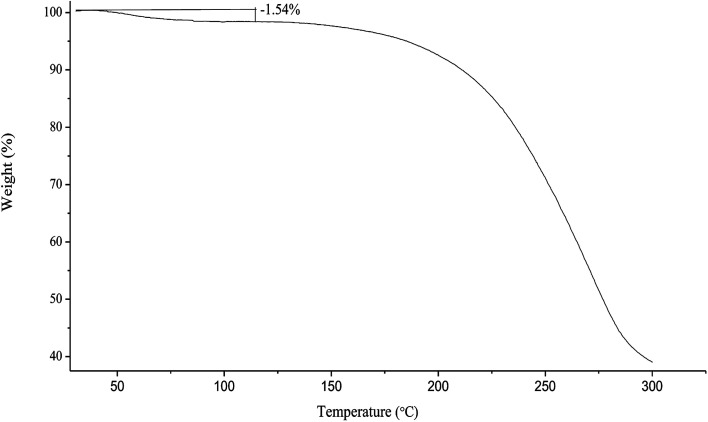
Thermogravimetric thermogram of spray dried chito-cubosomes.

Under heating on TGA, the spray-dried chito-cubosomes lose weight about 1.54% between 40 °C and 110 °C (Fig. 6), which was similar to the water content of 1.63% determined by V20 Karl Fischer Titrator (Mettler-Toledo AG, Greifensee, Switzerland), suggesting the initial decrease up to 110 °C was due to the removal of moisture. The rapid weight loss above 180 °C was attributed to the thermal degradation of chito-cubosomes.

### 3.3. Drug loading

The solubilities of VPT in GMO, water and buffer solution were shown in Table 1. It showed that VPT was poorly soluble in aqueous solution with the solubility lower than 5 μg mL^−1^, while it was easily dissolved in GMO with the solubility around 40 mg mL^−1^. The advantage of the liquid crystalline phase, as a drug delivery matrix, arises from its ability to significantly improve the solubility of VPT with a high SER value (around 10^5^), resulting in a high encapsulation efficiency of VPT in chito-cubosomes (>94%, Section 3.4). The determined VPT content in spray-dried chito-cubosomes was around 9.1 mg g^−1^.

**Table 1:** Solubilities of vinpocetine in different mediums (*n* = 3, mean ± S.D.)

Medium	Solubility (μg mL^−1^)	SER
GMO	40 500.5 ± 128.4	—
Pure water	4.2 ± 0.21	9643.0
pH 6.5 phosphate buffer	2.9 ± 0.13	13 965.7

### 3.4. Encapsulation efficiency

The encapsulation efficiency of VPT loaded in liquid cubosomes and spray-dried chito-cubosomes were determined to be 96.4% and 94.1%, indicating that almost all added VPT was encapsulated inside the cubosomes or chito-cubosomes. The high encapsulation efficiency of VPT could be attributed to its high lipophilicity (log *P*: 4.35, calculated by ALOGPS 2.1 program, http://www.vcclab.org/lab/alogps/) and its high solubility in lipid bilayers of cubosomes, which was similar to other highly lipophilic drugs loaded in cubosomes (*e.g.*, 98% for flurbiprofen,^23^ 98% for simvastatin and 86.2–92.4% for cyclosporine A^45^).

### 3.5. Influence of CS cross-linking on *in vitro* digestibility of cubosomes

*In vitro* digestion study was conducted to examine the influence of cross-linked CS on the digestibility of GMO based cubosomes. GMO is an ester synthesized from glycerol and oleic acid by esterification. After oral administration, GMO is easily hydrolyzed to free oleic acid by pancreatic lipase and bile salt. The rate and extent of such hydrolysis process of GMO could be simulated and quantified by *in vitro* pH-stat titration in artificial intestinal fluid that contains lipase.

**Fig. 7:**
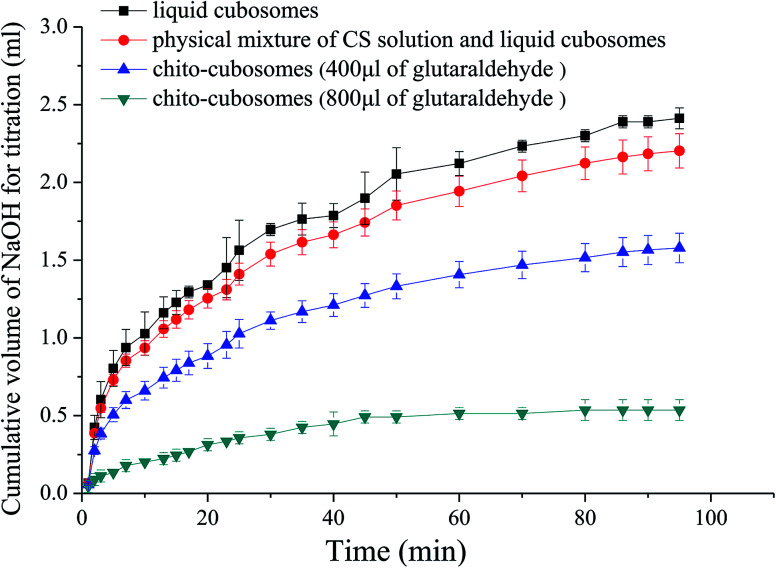
*In vitro* digestion profiles of liquid cubosomes, physical mixture of CS solution and liquid cubosomes, and chito-cubosomes using 400 μL or 800 μL of glutaraldehyde for cross-linking.

The titration profiles for VPT loaded liquid cubosomes and spray-dried chito-cubosomes are shown in Fig. 7. Previously reported studies suggested that such digestion of GMO after oral administration was responsible for the lack of sustained-release effect for cinnarizine. Cubosomes was rapidly digested as reflected by the large volume of titrant consumed. Without cross-linking, CS (*i.e.* physical mixture of CS and cubosomes in Fig. 7) has no obvious effect against the digestion of cubosomes. However, after cross-linking by glutaraldehyde, the obtained chito-cubosomes significantly inhibited the digestion of cubosomes by strengthening the cross-linking network.^46^ In addition, adding 800 μL of glutaraldehyde as crosslinking agent demonstrated significantly stronger anti-digestibility of chito-cubosomes than that using half volume of glutaraldehyde. Therefore, 800 μL of glutaraldehyde was used for the cross-linking of chito-cubosomes in this study. In the final obtained product (spray-dried chito-cubosomes), the residual glutaraldehyde was determined to be 0.005 mg g^−1^ according to HPLC method previously reported.^28^ The permitted daily exposure value (PDE) of glutaraldehyde *via* oral route was calculated to be 0.075 mg day^−1^ for human.^47–49^ Based on the maximum daily dosage of VPT (30 mg) and the drug content of chito-cubosomes (9.1 mg g^−1^), the calculated glutaraldehyde in maximum dosing was 0.016 mg day^−1^, which was much lower than the PDE.

### 3.6. *In vitro* release study

**Fig. 8:**
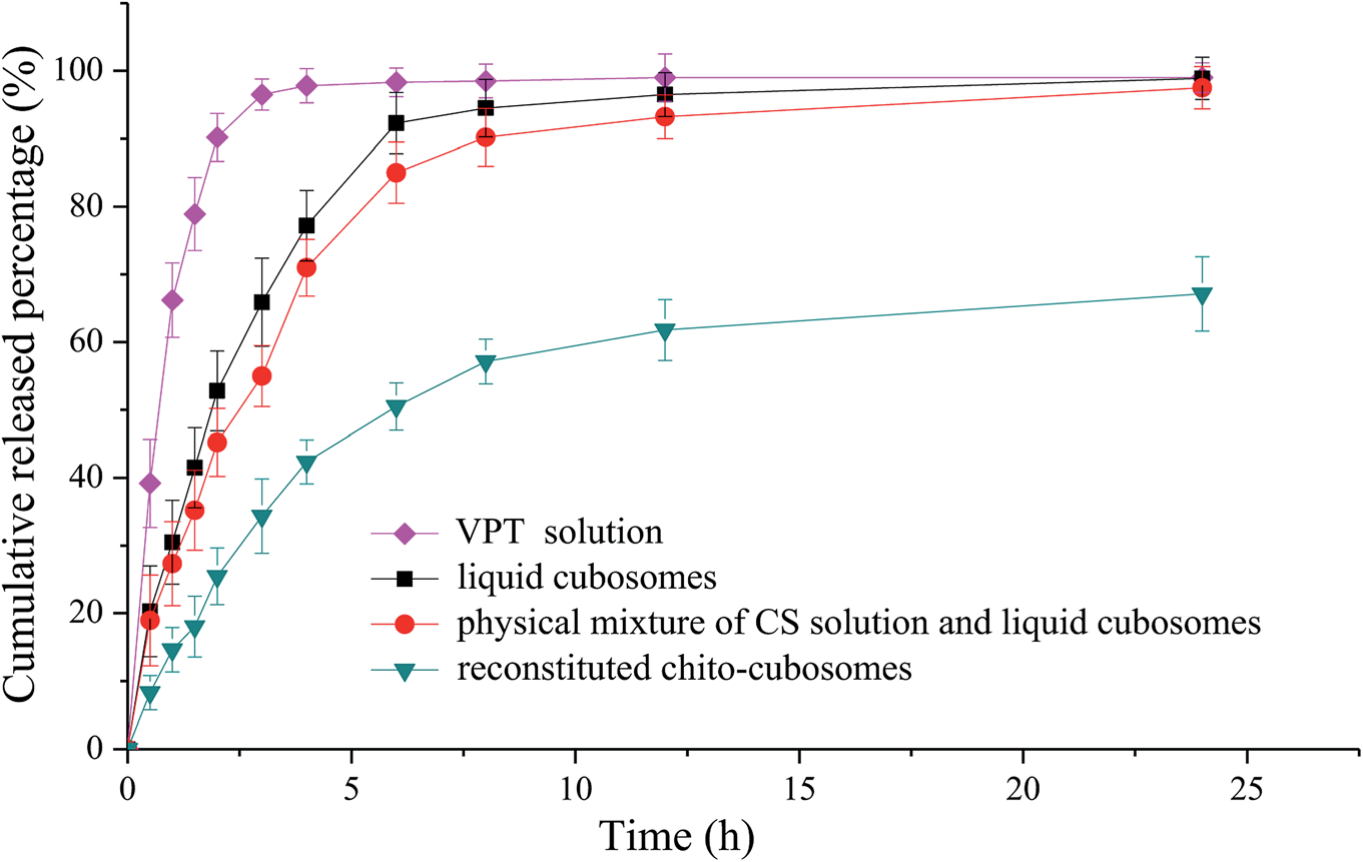
*In vitro* release profiles of VPT dialyzed from solution, liquid cubosomes, physical mixture of CS solution and liquid cubosomes, and reconstituted chito-cubosomes.

The *in vitro* release profiles of VPT from solution, liquid cubosomes, and spray-dried chito-cubosomes are shown in Fig. 8. The release of VPT from its solution in dialysis bag was very fast with the complete release achieved in 3 h. After loading in the cubosomes, significant decrease of drug release was observed with only 65% release at 3 h, such sustained release property of liquid cubosomes were also found when loading other highly lipophilic drug (*e.g.* simvastatin). After mixing liquid cubosomes with CS solution, the release of VPT showed a slight decrease, which might be due to the higher viscosity produced by CS in the system. However, after cross-linking with glutaraldehyde and spray drying, dramatic decrease in the release of VPT from reconstituted chito-cubosomes was observed. In comparison to untreated liquid cubosomes, only about half amount of VPT released from chito-cubosomes during the whole period of *in vitro* release study, which might be due to the reinforced surface structure of chito-cubosomes after cross-linking with glutaraldehyde.

### 3.7. *In vivo* absorption study

**Fig. 9:**
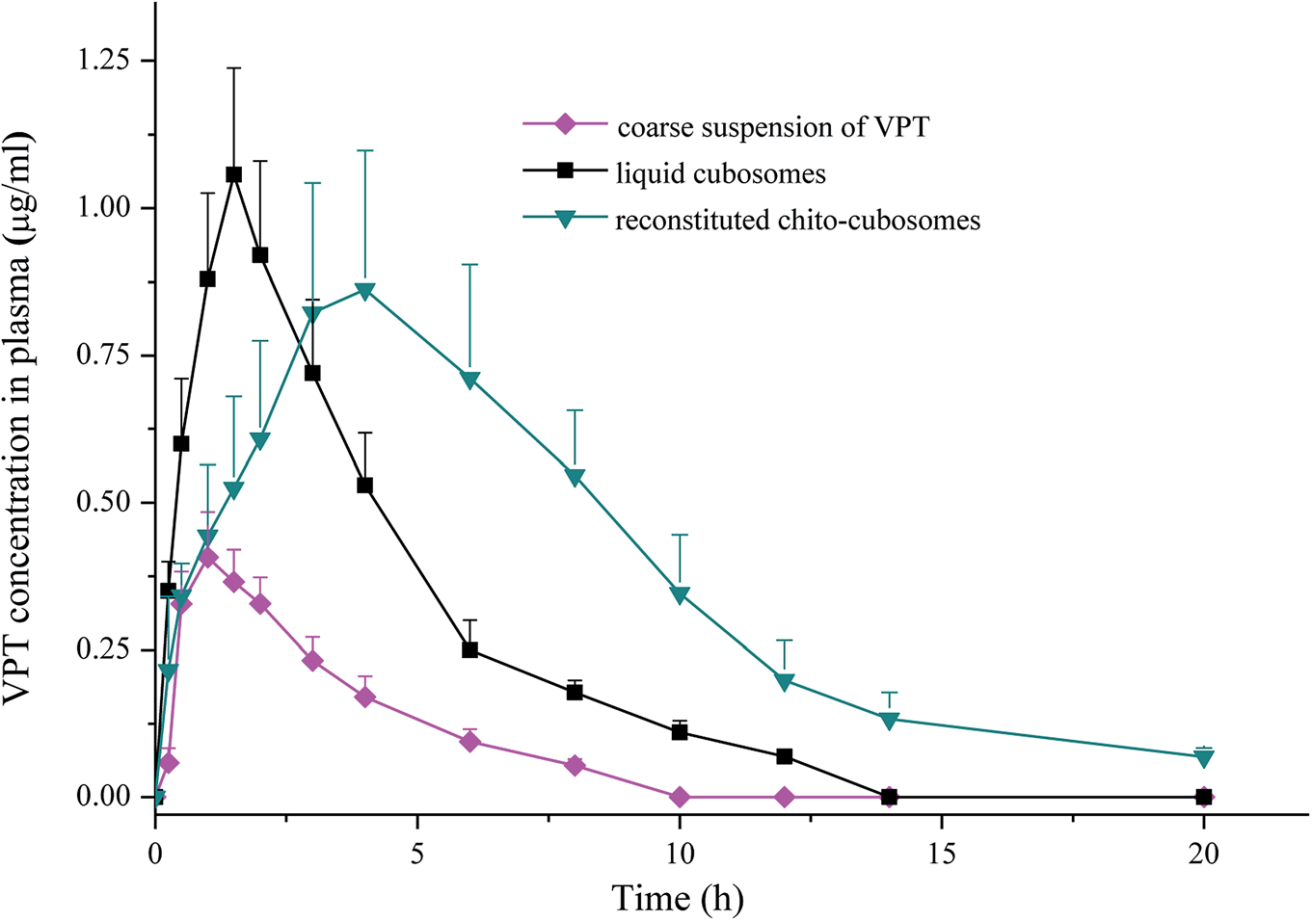
Plasma concentration *versus* time profiles of VPT in rats after single-dose oral administrations of coarse suspension of VPT, liquid cubosomes, and reconstituted chito-cubosomes. Each point represents mean ± S.D. (*n* = 5).

The mean plasma concentration *versus* time profiles of VPT after oral administration of liquid cubosomes and reconstituted spray-dried chito-cubosomes are shown in Fig. 9. Compared to coarse suspension of crystalline VPT, both liquid cubosomes and reconstituted chito-cubosomes showed significantly higher VPT plasma concentration profiles post administration. The calculated pharmacokinetic parameters are summarized in Table 2.

**Table 2:** Pharmacokinetic parameters of VPT after oral administration of coarse suspension of crystalline VPT, liquid cubosomes and chito-cubosomes at a single dose of 10 mg kg^−1^ (*n* = 5, mean ± S.D.)

Parameters	Suspension of VPT	Liquid cubosomes	Chito-cubosomes
*T*_max_ (h)	1.28 ± 0.05	1.52 ± 0.12^a^	3.57 ± 0.18^b^^,^^c^
*C*_max_ (μg ml^−1^)	0.38 ± 0.06	0.96 ± 0.12^a^	0.80 ± 0.09^b^
AUC _0–20 h_ (μg ml^−1^ h)	1.47 ± 0.12	4.53 ± 0.25^b^	7.59 ± 0.32^b^^,^^c^
AUC _0–∞_ (μg ml^−1^ h)	1.56 ± 0.17	4.62 ± 0.31^b^	7.78 ± 0.55^b^^,^^c^
MRT (h)	3.22 ± 0.89	3.70 ± 0.77	7.14 ± 1.25^b^^,^^c^
RB (%)	N.A.	296.1	498.7%

a *p* < 0.05 *versus* suspension of VPT;

b *p* < 0.01 *versus* suspension of VPT;

c *p* < 0.01 *versus* liquid cubosomes;

In comparison to coarse suspension, *C*_max_ and AUC_0–∞_ of VPT were significantly enhanced after oral administration of liquid cubosomes by 2.5-fold and 3.0-fold, respectively. Several factors could be involved in the improvement of the oral bioavailability of VPT. Um *et al.* investigated the absorption mechanism of cubosomes by the digestive tract using Caco-2 cells and rat jejunum, and found both released drugs (normally by transcellular and/or paracellular transport) and drug embedded in cubosomes (by endocytosis) could transport across the endothelial cell membranes, thus achieving enhanced drug absorption.^50^ In addition, cubosomal structure would be solubilized by bile salt in small intestine into mixed micelles, which acts as lyotropic carriers with hydrophilic surface which facilitates the contact with the endothelial cell membrane by overcoming the “unstirred water layer” barrier.^51–53^ On the other hand, although liquid cubosomes significantly enhanced the absorption of VPT, the rapid digestion of GMO and the subsequent collapse of liquid crystalline matrix resulted in fast release of drug from cubosomes, preventing the maintenance of sustained release. This effect was supported by the observation that the *T*_max_ and MRT of VPT were not significantly affected by administration of VPT-loaded liquid cubosomes (Table 2).

In comparison to liquid cubosomes, the spray-dried chito-cubosomes showed significantly higher AUC_0–∞_ (1.7-fold) with slightly lower *C*_max_ of VPT (Table 2). The relative bioavailability of chito-cubosomes was calculated to be 498.7% using coarse suspension of VPT as control. The further enhanced bioavailability might be attributed to the presence of muco-adhesive CS in the spray-dried chito-cubosomes. As a natural biodegradable polymer with positive charges, it is well-known that CS could act as a penetration enhancer for drugs by temporary widening of the paracellular route, thus enhancing their *in vivo* absorption.^54,55^ In addition, the bioadhesive behavior of CS would prolong the residence time of the formulation on the intestinal epithelium surface so as to increase the absorption after oral administration.^56,57^ In addition, chito-cubosomes demonstrated a 2.3-fold delayed *T*_max_ and a 1.9-fold longer MRT in comparison to liquid cubosomes (Table 2), exhibiting a sustained absorption behavior, which might be due to its *in vitro* sustained release behavior caused by protection from lipid digestion of GMO *via* CS cross-linking (Fig. 8).

## 4. Conclusion

In this study, a spray-dried VPT cubosomes with surface cross-linked CS were prepared and characterized. In comparison to the coarse suspension of crystalline VPT, GMO based liquid cubosomes showed significantly higher oral absorption of VPT. After subsequent surface modification with cross-linked CS, the obtained chito-cubosomes exhibited anti-digestion effect, much slower *in vitro* release behavior, and further enhanced oral bioavailability than intact liquid cubosomes. Our results suggested that the chito-cubosomes would be a potential carrier for sustained delivery of highly lipophilic drugs with low oral bioavailability.

## Conflicts of interest

The authors declare no competing financial interest.

## Supplementary Material
